# Designing and validating an experimental protocol to induce airway narrowing in older adults with and without asthma

**DOI:** 10.1186/s12938-023-01197-6

**Published:** 2024-03-06

**Authors:** Shaghayegh Chavoshian, Xiaoshu Cao, Anirudh Thommandram, Matthew B. Stanbrook, Susan M. Tarlo, Yan Fossat, Azadeh Yadollahi

**Affiliations:** 1 KITE Research Institute, Toronto Rehabilitation Institute, University Health Network, 550 University Ave, Toronto, M5G 2A2 ON Canada; 2 Institute of Biomedical Engineering, University of Toronto, 164 College St., Toronto, M5S 3G9 ON Canada; 3 Labs Department, Klick Health, Klick Inc, 175 Bloor St. East, Toronto, M4W 3R8 ON Canada; 4 Department of Medicine, University of Toronto, 164 College St., Toronto, M5S 3G9 ON Canada; 5 Department of Medicine, University Health Network Toronto Western Hospital, 399 Bathurst St., Toronto, M5T 2S8 ON Canada; 6 Dalla Lana School of Public Health, University of Toronto, 155 College St., Toronto, M5T 1P8 ON Canada

**Keywords:** Asthma, Airway narrowing, Cold temperature, Exercise, Experimental protocol

## Abstract

**Background:**

Persons with asthma may experience excessive airway narrowing due to exercise or exposure to cold air, worsening their daily functionality. Exercise has several benefits for asthma control, but it may induce airway narrowing in some persons with asthma. When combined with cold temperatures, it introduces another layer of challenges. Therefore, managing this interaction is crucial to increase the quality of life in individuals with asthma. The purpose of this study was to develop a reliable experimental protocol to assess the effects of exercise and cold air on airway narrowing in adults with asthma in a controlled and safe environment.

**Methods:**

This study was a randomized cross-over study in adults with and without asthma. Participants underwent a protocol involving a 10-min seated rest, followed by a 10-min cycling on a stationary bike in different temperatures of 0, 10, or 20 ^∘^C. The sequence of room temperatures was randomized, and there was a 30-min interval for recovery between each temperature transition. In each temperature, to measure lung function and respiratory symptoms, oscillometry and a questionnaire were used at 0 min (baseline), after 10 min of sitting and before starting biking (pre-exercise), and after 10 min of biking (post-exercise). At each room temperature, the changes in airway mechanics and asthma symptoms among baseline, pre-exercise, and post-exercise were compared with one-way repeated measures ANOVA or Friedman Rank Test. Within each arm, cardiac and thoraco-abdominal motion respiration signals were also measured continuously using electrodes and calibrated respiratory inductance plethysmographs, respectively.

**Results:**

A total of 23 persons with asthma (11 females, age: 56.3 ± 10.9 years, BMI: 27.4 ± 5.7 kg/m^2^) and 6 healthy subjects (3 females, age: 61.8 ± 9.1 years, BMI: 28.5 ± 3.1 kg/m^2^) were enrolled in the study. Cold temperature of 0 ^∘^C induced airway narrowing in those with and without asthma after 10 and 20 min, respectively. Exercise intervention had significant changes in airway narrowing in participants with asthma in the range of 10–20 ^∘^C. Our results showed that in asthma, changes in subjective respiratory symptoms were due to both cold temperatures of 0 and 10 ^∘^C and exercise in the 0–20 ^∘^C range. Respiratory symptoms were not noticed among the healthy participants.

**Conclusion:**

In conclusion, our findings suggest that exposure to cold temperatures of 0 ^∘^C could serve as a reliable method in the experimental protocol for inducing airway narrowing in asthma. The impact of exercise on airway narrowing was more variable among participants. Understanding these triggers in the experimental protocol is essential for the successful management of asthma in future studies.

## Introduction

Asthma is an inflammatory disease of the airways that can make breathing difficult [1, 2]. There are a variety of factors in asthma that can lead to respiratory insufficiency by inducing airway narrowing [2, 3]. Persons with asthma may experience wheezing, dyspnea, shortness of breath, and chest tightness resulting in limited daily activities, hospitalization, morbidity, and mortality [1, 4–6]. Asthma is associated with 1 out of every 250 fatalities globally, and a significant portion of these cases involve individuals aged 45 or older [5, 7]. The burden of asthma impacts the economy of a country, with 1–2% of the overall healthcare expenses in developed countries [7–10].

Asthma has no cure, but it can be controlled [11]. It has been reported that exercise can improve asthma symptoms and overall respiratory health [12, 13]. In turn, physical activity, specifically in cold temperatures can be a risk factor for airway inflammation and excessive airway narrowing in asthma [2, 4]. Although pre-treatment with a bronchodilator/inhaled steroid is a common clinical recommendation, approximately 90% of asthma cases develop post-exercise symptoms [14, 15]. Given that, individuals may opt to avoid physical activities. The results suggest that cold weather may impact the outdoor exercise that older persons are able to perform during the winter months. Therefore, a better understanding of the effects of exercise and cold weather on asthma may bring new approaches to reducing the burden of asthma.

In the course of studying patients with asthma, significant changes in lung function were demonstrated after breathing or exercising in cold air ranging from − 8 to − 15 ^∘^C for an average of 3.4 min [16]. An earlier study showed that cycling in dry cold air for 9 min caused a greater level of bronchoconstriction compared to cycling in warm and more humid air. It was also concluded that cycling and running can induce airway narrowing similarly [17]. An experimental study indicated that respiratory resistance obtained from the impulse oscillometry system increased significantly after cycling for 6 min at − 1 ^∘^C, while these changes were not detectable by spirometry [18]. There is evidence from another study indicating that the forced oscillation technique (FOT) is a sensitive measurement for airway narrowing due to exercise [19]. While several studies have investigated the mechanisms underlying cold or exercise-induced asthma, current protocols are limited in age range variability, precise temperature or exposure duration, understanding mechanisms in the experimental setting, and comparing these two triggers within the same study [16, 20–25]. Therefore, further investigation is needed.

It is not clear what types of exercise or temperature challenges, or their combination, may be an effective test to induce airway narrowing in persons with asthma, while remaining safe and achievable for them, especially for older adults who may have more restricted exercise capabilities. The present study was carried out to develop an experimental protocol to induce airway narrowing in a safe and reproducible way. We investigated two interventions, exercise and cold temperature in middle-aged and senior adults with and without asthma.

## Results

In this randomized cross-over study, we asked adults with and without asthma to sit for 10 min followed by cycling on a stationary bike for 10 min in various room temperatures of 0, 10, or 20 ^∘^C. The sequence of room temperatures was randomized, and there was a 30-min interval for recovery between each temperature transition. Before and after each study arm, to measure lung function and respiratory symptoms, we used FOT and the Borg scale questionnaire, respectively. Electrocardiograms and thoraco-abdominal respiratory movements were recorded continuously during the experiment. At each room temperature, the changes in lung function and respiratory symptoms were compared using statistical tests.

### Demographics of participants

A total of 6 healthy individuals and 23 individuals with asthma were enrolled in the study. The demographics, questionnaire indices, and baseline values of lung function for both groups of healthy and asthma are reported in Table 1. There were no significant differences between the healthy and asthma groups in sex, age, height, weight, and body mass index (BMI). We assessed the level of asthma control by the Asthma Control Test (ACT), and a score above 19 indicates controlled asthma [26, 27]. For the participants with asthma, the ACT score was 22.3 ± 3.8 indicating that the asthma level in the participants in this study was controlled. Exercise tolerance, activity level, and functional status were assessed using the Duke Activity Status Index, Leisure Index of Baecke Physical Activity, and Functional Outcomes of Sleep Questionnaire (FOSQ-10), respectively. Based on the results of these questionnaires, there were no significant differences between asthma and healthy participants.

### Forced oscillation technique (FOT)

We used FOT to measure lung function. The primary outcomes were resistance at 5 Hz (*R*_5_), reactance at 5 Hz (*X*_5_), area of reactance (*A*_x_), and resonance frequency (F_res_). These parameters represent total respiratory resistance, elastic recoil of the peripheral airways, peripheral airway narrowing, and the frequency at which the airflow impedance is entirely resistive to flow, respectively [28, 29]. The baseline values of *R*_5_, *X*_5_, *A*_x_, and *F*_res_ were not significantly different between asthma and healthy groups, potentially due to the small number of healthy individuals and their older age.

**Fig. 1:**
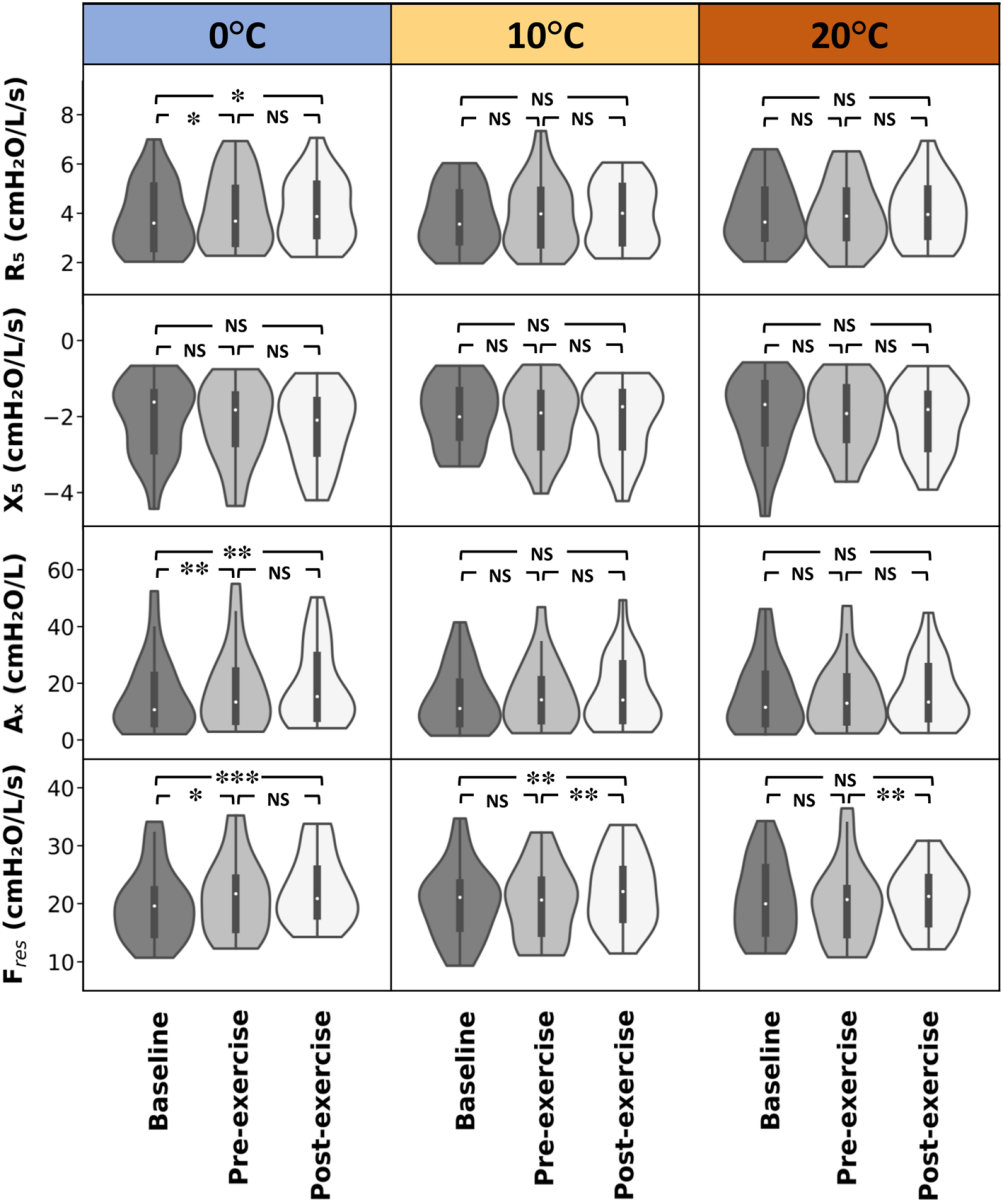
Respiratory system parameter changes from baseline to post-exercise in temperatures of 0, 10, and 20 ^∘^C within the asthma group. Demonstrated p values in the figure show the post-hoc analysis which is either Tukey HSD tests or pairwise signed-ranks tests. *R*_5_, respiratory system resistance at 5 Hz; *X*_5_, respiratory system reactance at 5 Hz; *A*_x_, reactance area, and *F*_res_, resonance frequency. *Represents $0.01< p < 0.05$, ** $0.0001< p < 0.01$, and *** $p < 0.0001$ for comparison between study arms

In temperature of 0 ^∘^C, there were significant increases in *R*_5_, *A*_x_, and *F*_res_ in the asthma group ($p = 0.02$, $p = 0.004$, and $p < 0.0001$, respectively). In participants with asthma at the temperature of 0 ^∘^C, there were significant changes in R_5_, A_x_, and F_res_ from baseline to pre-exercise ($p = 0.04$, $p = 0.003$, and $p = 0.02$, respectively) and baseline to post-exercise ($p = 0.03$, $p = 0.001$, and $p < 0.0001$, respectively). However, in healthy participants at 0 ^∘^C, only A_x_ increased significantly from baseline to post-exercise ($p = 0.03$). At 0 ^∘^C, there was no significant change in *X*_5_ in either group. In temperature of 10 ^∘^C, for the asthma group, only *F*_res_ increased significantly from baseline and pre-exercise to post-exercise ($p = 0.005$, $p = 0.004$, respectively). Conversely, there were no significant differences in *R*_5_, *X*_5_, *A*_x_, and *F*_res_ in the healthy group at 10 ^∘^C. In the temperature of 20 ^∘^C, for the asthma group, only *F*_res_ increased significantly from pre-exercise to post-exercise ($p = 0.02$, one subject was excluded for non-recorded *F*_res_). In the healthy group, there were no changes in *R*_5_, *X*_5_, *A*_x_, and *F*_res_ in 20 ^∘^C (Fig. 1).

### Subjective asthma symptoms

**Fig. 2:**
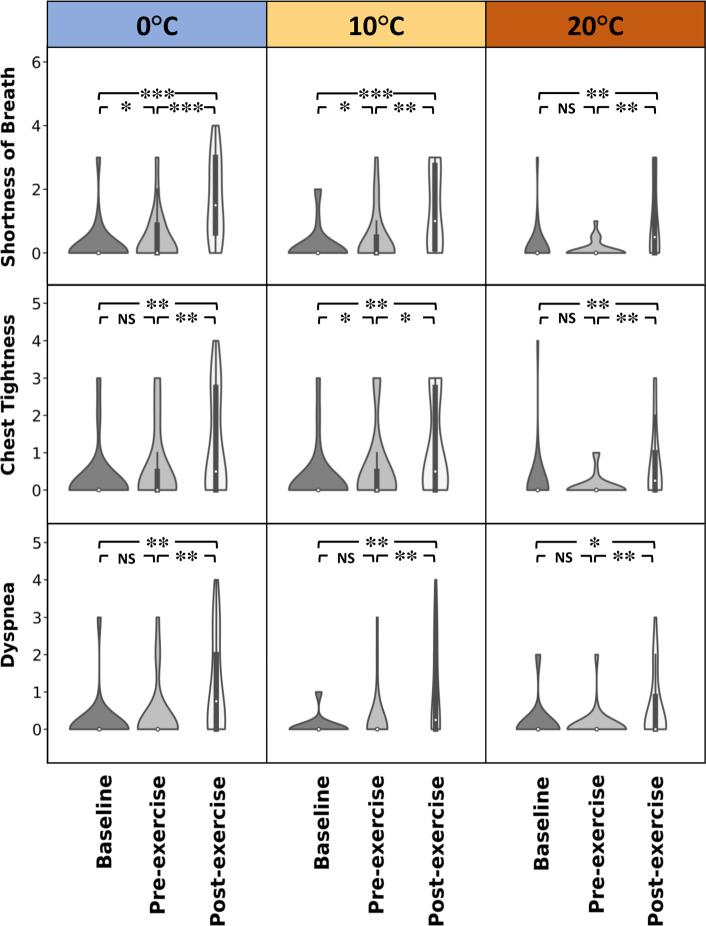
Subjective asthma symptoms changes from baseline to post-exercise in temperatures of 0, 10, and 20 ^∘^C within the asthma group (22 participants out of 23). Demonstrated *p* values in the figure show the post-hoc analysis which is either Tukey HSD tests or pairwise signed-ranks tests. *Represents $0.01< p < 0.05$, **$0.0001< p < 0.01$, and ***$p < 0.0001$ for comparison between arms

The Modified Borg scale was used for the subjective assessment of shortness of breath, chest tightness, and dyspnea [30, 31]. In 22 individuals with asthma (one subject’s outcome was missing), in all three temperatures, there were significant increases in the Borg scale for shortness of breath, chest tightness, and dyspnea from baseline to post-exercise and from pre- to post-exercise. Furthermore, shortness of breath increased significantly from baseline to pre-exercise at 0 and 10 ^∘^C. Chest tightness increased from baseline to pre-exercise only at 10 ^∘^C (Fig. 2). In healthy individuals, the average of all Borge scores was 0 and did not change due to exercise or room temperature changes.

### Exercise intensity based on cardiac output

Maximal heart rate achieved during exercise is often used to categorize exercise intensity. The target maximal heart rate is calculated by subtracting age from 220 [32]. We also calculated heart rate using the recorded cardiac signal for each participant. Consequently, light, moderate, and vigorous exercise intensity were defined as heart rate $< 65$%, 64–76%, and 77–93% of the maximum heart rate, respectively [33]. The level of exercise intensity varied among participants for different temperatures. Table 2 depicts the number of participants categorized by exercise intensity.

**Table 1:** Participants’ baseline characteristics (mean ± SD)

Characteristics	Healthy ($n=6$)	Asthma ($n=23$)	*p* value
Sex (male/female)	3/3	12/11	Chi^2^, $p = 0.92$
Age, years	61.8 ± 9.1	56.3 ± 10.9	0.27
Height, cm	163.5 ± 10.8	166.4 ± 10.2	0.54
Weight, kg	76.0 ± 10.4	75.7 ± 15.9	0.96
BMI, kg/m^2^	28.5 ± 3.1	27.4 ± 5.7	0.31
ACT	–	22.3 ± 3.8	–
Duke Activity Status Index	47.3 ± 15.3	48.6 ± 12.1^a^	0.97
Baecke Physical Activity-Leisure Index	2.9 ± 0.3	2.8 ± 0.8^a^	0.93
FOSQ-10 Score	17.4 ± 2.4	17.9 ± 2.5^a^	0.56
R_5_, cmH_2_O/L/s	3.5 ± 1.4	3.9 ± 1.2	0.48
X_5_, cmH_2_O/L/s	− 1.7 ± 1.1	− 2.1 ± 1.1	0.57
A_x_, cmH_2_O/L	11.1 ± 11.3	15.9 ± 12.4	0.37
F_res_, cmH_2_O/L/s	17.5 ± 6.9	20.6 ± 6.4	0.31

*p* values are based on unpaired t-test, Wilcoxon test, or chi-square test

^a^For these questionnaires, the number of participants with asthma was 21 due to missing values

*SD,* standard deviation; *BMI,* body mass index; *ACT,* Asthma Control Test; *R*_5_, respiratory system resistance at 5 Hz; *X*_5_, respiratory system reactance at 5 Hz; *A*_x_, reactance area; *F*_res_, resonance frequency

**Table 2:** Distribution of exercise intensity across groups and interventions

Group	Intervention	Exercise intensity (%)		
		Light	Moderate	Vigorous
Asthma	Exercise in 0 ^∘^C	13% (*n* = 3)	48% (*n* = 11)	39% (*n* = 9)
	Exercise in 10 ^∘^C	35% (*n* = 8)	43% (*n* = 10)	22% (*n* = 5)
	Exercise in 20 ^∘^C	56% (*n* = 13)	35% (*n* = 8)	9% (*n* = 2)
Healthy	Exercise in 0 ^∘^C	33% (*n* = 2)	33% (*n* = 2)	33% (*n* = 2)
	Exercise in 10 ^∘^C	33% (*n* = 2)	50% (*n* = 3)	17% (*n* = 1)
	Exercise in 20 ^∘^C	50% (*n* = 3)	33% (*n* = 2)	17% (*n* = 1)

The percentages represent the proportion of participants in each exercise intensity category. The numbers in parentheses (*n*) indicate the corresponding participant count

## Discussion

In this study, we developed a novel experimental protocol to combine cold temperature and exercise for the assessment of lung function in older adults with and without asthma. Based on objective and subjective assessments, our main findings include the following. (1) Based on objective assessment, in individuals with asthma, exposure to cold temperature was more effective than exercise to induce airway narrowing. (2) Based on subjective assessment, in individuals with asthma, respiratory symptoms worsen due to both cold temperature and exercise. These novel findings could help to develop more robust and reproducible experimental protocols to assess lung function in asthma. (3) In healthy individuals, while objective assessment indicated that exercise at 0 ^∘^C for 20 min induced airway narrowing, there were no changes in subjective assessment.

Based on FOT results, we found that exposure to cold temperature of 0 ^∘^C may be a more robust intervention to induce airway narrowing in persons with asthma, compared to exercise. However, resonance frequency changes in 10 and 20 ^∘^C were significant due to exercise. Increased resonance frequency reflects more airway obstruction [34]. On the other hand, subjective results showed that respiratory symptoms were increased in 0 and 10 ^∘^C significantly due to both cold temperature and exercise in cold. In 20 ^∘^C, participants with asthma reported worsening of symptoms only after exercise. Prior studies demonstrated that exposure to or exercise in cold temperature can cause shortness of breath and increased production of lung mucus in individuals with asthma [35–38]. This was also verified in other studies that demonstrated more chest tightness and dyspnea with exercise in those with asthma [38, 39]. It is worth noting that objective outcomes may be more reliable as other feelings such as fatigue or increased breathlessness after exercise may impact the subjective results [40]. The discrepancy between subjective and objective measures could be because participants with asthma did not exercise hard enough in temperatures of 0 ^∘^C, as they felt shortness of breath before there were changes in objective measures. Therefore, they were concerned about asthma attacks that stopped them from exercising.

In the asthma group, the scores for the Duke Activity Status Index, Leisure Index of Baecke Physical Activity, and FOSQ-10 were high, indicating a good level of exercise tolerance, activity level, and functional status. However, we observed that the distribution of participants’ exercise intensity changed with temperature. The percentage of participants with vigorous-intensity exercise was higher at the temperature of 0 ^∘^C. Conversely, exercise did not have significant effects on airway narrowing changes in this temperature. Therefore, the observed higher number of vigorous-intensity instances may not solely be attributed to the exercise itself, but could be influenced by the cold temperature conditions, increasing heart rate, and perceptions of exertion. This observation is aligned with previous studies that indicated cold temperature effects on increasing heart rate [41, 42].

The significant increase in airway narrowing in the asthma group due to cold temperature of 0 ^∘^C is consistent with previous findings [43]. In a longitudinal investigation among adults with asthma, the findings revealed a significant association between asthma exacerbation and cold temperatures ranging from − 7 to 3 ^∘^C [43]. Cold temperature is highly associated with hospitalizations and emergency department visits due to asthma [44, 45]. Most of the prior studies are based on observational data and lack controlled settings. Thus, the results may be influenced by other confounding factors such as winter inversions and temperature drops [46, 47]. Consequently, in addition to aligning with previous findings, our results contributed to a more robust conclusion about the impact of temperature on airway narrowing in asthma. On the other hand, during exercise, faster and deeper breathing through the mouth leads to inhaling dry and cool air, which can cool pulmonary blood and stimulate pulmonary stretch receptors [20, 23, 24]. As a result, it increases airway smooth muscle contractions and mucus accumulation causing airway narrowing [21]. Airway constriction may also occur due to facial cooling [22, 48].

Based on baseline objective measurements, there were no changes between the two groups of healthy and asthma. A possible explanation for this observation is the small sample size of healthy participants. It also could be because of the effects of the older age of participants without asthma. Similar to the results of [37], we found that healthy participants only had significant increases in small airway narrowing from baseline to after exercise in 0 ^∘^C. Fontanari et al. also showed in healthy participants as well as those with asthma, inhaling cold air can cause airway narrowing [49].

In this study, individuals with asthma were more sensitive to airway narrowing due to cold temperature and exercise compared to healthy subjects. These findings align with the results of studies indicating that the effects of cold and exercise are aggravated in persons with respiratory disorders such as asthma compared to healthy individuals [20, 50–52].

The present study has some limitations. In our study, subjects were instructed to exercise at the maximally comfortable pace to not put them at any risk. Therefore, the changes in respiratory impedance have not been detected in all participants. Our study was also limited to measuring the exercise load which was likely less when the room temperature was lower. The study sample size was rather small. Approximately 10% of patients with asthma who were contacted, denied participating in the study as they had hospitalization experiences due to either cold or exercise stimuli.

## Conclusion

In conclusion, we found that compared to exercise, exposure to cold temperatures is a more reliable and practical protocol to induce airway narrowing in asthma. We also found that both objective and subjective measures are required to have a better understanding of the burden of exercise and cold temperature on airway narrowing and lung function in asthma. Once validated in a larger and more diverse sample size, the findings of this study may help to design safe and effective strategies to assess asthma symptoms triggered by cold exposure and exercise. In the future, this experimental protocol can be used to assess physiological monitoring specifically electrocardiogram and respiration signals to estimate airway narrowing during everyday activities specifically in cold weather in persons with asthma.

## Methods

### Study participants

Healthy participants were recruited by advertisement and participants with asthma were recruited from Toronto Western Hospital asthma clinic. Asthma was diagnosed based on Global Initiative for Asthma (GINA) guidelines [53]. The inclusion criteria for both healthy and asthma groups were non-smoker adults in the age range of 18–80 years. The exclusion criteria included pregnancy, any cardiovascular or renal disorders, other severe lung diseases, or use of any medication for these conditions, uncontrolled hypertension, or stage 2 hypertension (systolic at least 140 mmHg or diastolic at least 90 mmHg). The eligibility for enrollment was determined by reviewing patient charts, and the study purpose and protocol were prepared for those who agreed to be contacted. The study protocol was approved by the Research Ethics Board at University Health Network, and all participants provided written informed consent prior to participation in the study.

### Lung function measurement

FOT is a non-invasive and passive method to measure respiratory mechanics [54]. In the present study, FOT measurements were acquired over 20 s and repeated for three acceptable and repeatable tests over a range of frequencies from 5 to 37 Hz. *R*_5_, *X*_5_, *A*_x_, and *F*_res_ were measured using TremoFlo FOT (Thorasys).

### Questionnaires

To assess the severity of asthma control, participants completed the ACT [26]. An ACT score greater than 19 shows controlled asthma [27]. Modified Borg shortness of breath/chest tightness/dyspnea scales yield a score on a scale from 0 to 10, in which a score of 10 is an indicator of very severe shortness of breath/chest tightness/dyspnea respectively. Functional capacity or exercise tolerance was assessed using the Duke Activity Status Index which is a 12-item questionnaire. The total score ranges from 0 to 58.2, with higher scores indicating better functional capacity [55]. To assess activity level, Baecke physical activity questionnaire was conducted. The questionnaire includes questions about work-related activities, sports, and leisure time activities, over a time period of 1 year. Given that most of our participants were retired or not actively engaged in sports, we only assessed the leisure index. The leisure index score is on a scale of 1–5 and a score of 5 indicates the most activity [56]. Functional status and quality of life were measured by the short version of FOSQ-10 with a possible range of 5–20. A higher total score indicates better functional outcomes [57].

### Protocol

**Fig. 3:**
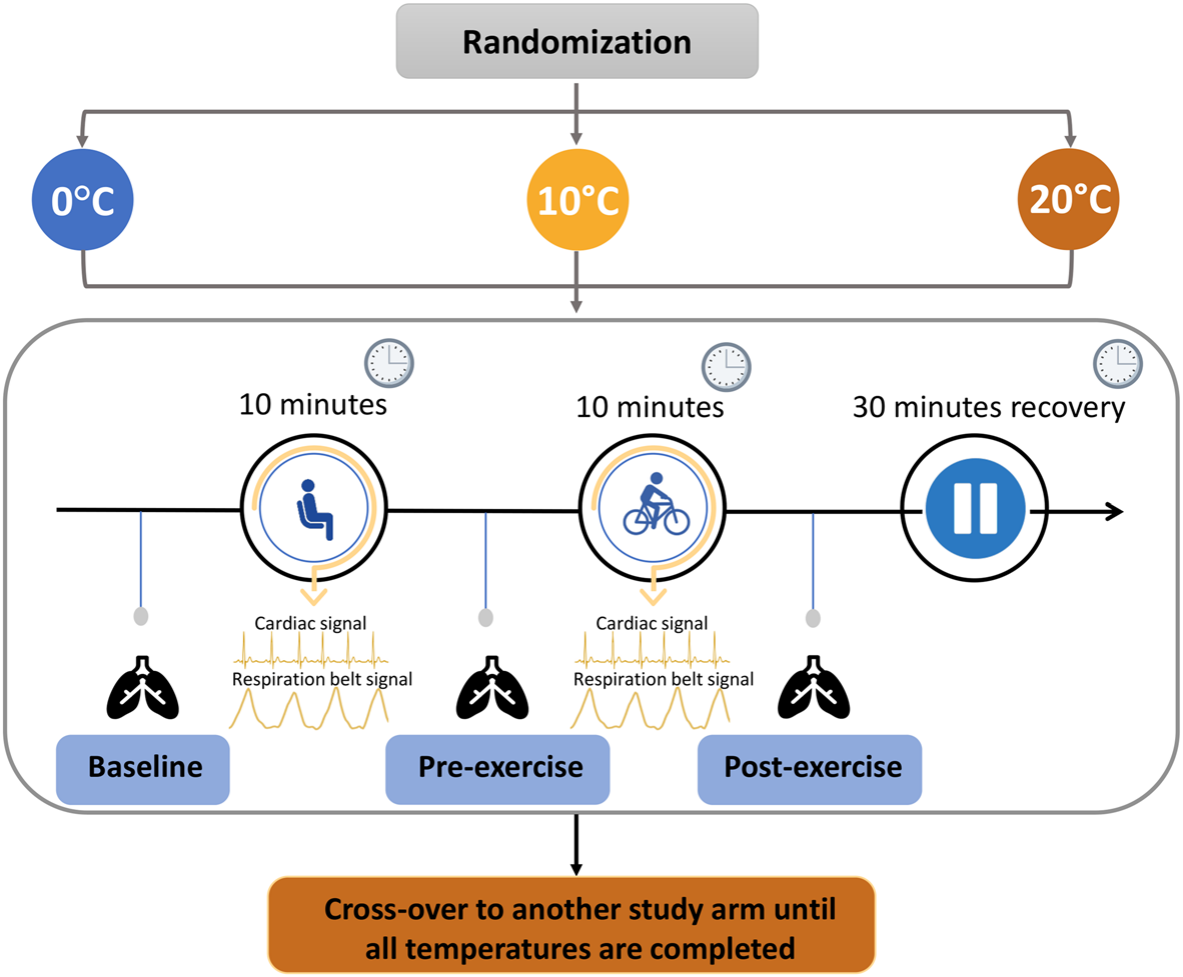
Randomized cross-over study of sitting for 10 min followed by cycling on a stationary bike for 10 min in various room temperatures of 0, 10, and 20 ^∘^C

Participants attended the climate laboratory located in the Toronto Rehabilitation Institute for a daytime study. Prior to the study measurements, participants were consulted by their respirologists to withhold asthma medications as follows: (1) short-acting $\beta_2$ agonists for 6 h, (2) long-acting $\beta_2$ agonists for 24 h, and (3) long-acting anticholinergic agents for 72 h. Participants first had their weight and height measured. The protocol was a randomized cross-over study, and interventions included exercise and changes in the room temperature. In the climate laboratory, temperature can be adjusted from − 20 ^∘^C to + 35 ^∘^C. Study participants were asked to sit for 10 min followed by 10 min of biking on a stationary bike while seated. The participants were asked to exercise at the maximum rate that they felt comfortable and could continue for 10 min. The room temperature was randomly set at either 0, 10 or 20 ^∘^C. After completing the protocol in each room temperature, there was a 30-min break for recovery and then participants repeated the protocol in another room temperature until they had been exposed to all three temperatures (Fig. 3). Within each arm, cardiac or electrocardiogram signal and thoraco-abdominal motion/respiration belt signals were measured continuously using electrodes and calibrated respiratory inductance plethysmographs, respectively. Participants performed all study arms on the same day, and they had adequate warm clothes such as winter jackets and gloves for cold temperatures. While seated, FOT was performed to measure the impedance of the respiratory system at 0 min (baseline), after 10 min of sitting and before starting biking (pre-exercise), and after 10 min of biking (post-exercise). The Modified Borg scale was also measured before and after each intervention.

### Statistical analysis

Values for demographic data, scores of questionnaires, baseline *R*_5_, baseline *X*_5_, baseline *A*_x_, and baseline *F*_res_ were compared between healthy and asthma participants using either a t-test or Wilcoxon Rank Sums test. Between-group changes in the lung function and Modified Borg scale were compared from baseline to post-exercise with a one-way repeated measures ANOVA or Friedman Rank Test. For post-hoc analysis, Tukey HSD tests or pairwise signed-ranks tests were conducted, respectively. Statistical significance was determined at $p \le 0.05$. We performed the statistical analyses in JMP package software which is developed by the SAS institute.

## Data Availability

The datasets generated and analyzed during the current study are not currently available to public, as they are not de-identified and the current research ethics approval does not allow the sharing of data. In the future, we will try to receive approvals to de-identify the data and make it available.

## Abbreviations

FOT: Forced Oscillation Technique
BMI: Body mass index
ACT: Asthma control test
FOSQ-10: Functional Outcomes of Sleep Questionnaire
*R*_5_: Resistance at 5 Hz
*X*_5_: Reactance at 5 Hz
*A*_x_: Area of reactance
*F*_res_: Resonance frequency
SD: Standard deviation
GINA: Global Initiative for Asthma
